# Meta-Analysis of Prevalence and Risk Factors for Cognitive Decline and Improvement After Transcatheter Aortic Valve Implantation

**DOI:** 10.1016/j.amjcard.2020.04.023

**Published:** 2020-07-15

**Authors:** Erica S. Ghezzi, Tyler J. Ross, Daniel Davis, Peter J. Psaltis, Tobias Loetscher, Hannah A.D. Keage

**Affiliations:** a Cognitive Ageing and Impairment Neurosciences Laboratory, Justice and Society, University of South Australia, Adelaide, Australia; b MRC Unit for Lifelong Health and Ageing Unit at UCL, London, United Kingdom; c Vascular Research Centre, Lifelong Health Theme, South Australian Health and Medical Research Institute, Adelaide, Australia; d Adelaide University Medical School, University of Adelaide, Adelaide, Australia; e Department of Cardiology, Central Adelaide Local Health Network, Adelaide, Australia

## Abstract

Changes to cognition, both decline and improvement, are commonly reported after transcatheter aortic valve implantation (TAVI). However, previous systematic reviews and meta-analyses have missed these subgroups by assessing whole-group-averages for cognitive outcomes. We sought to pool estimates to identify the prevalence of cognitive decline and improvement after TAVI, as well as associated factors for these outcomes. A systematic review identified 15 articles appropriate for meta-analysis. When robust cognitive change definitions were employed, the pooled prevalence of incident cognitive impairment up to 1-, 1 to 6-, and ≥6-months post-TAVI was 7%, 14%, and 12%, respectively. For cognitive improvement, the prevalence from 1 to 6 months and ≥6 months after TAVI was estimated to be 19% and 11%, respectively. Two factors were associated with these cognitive outcomes: (1) using a cerebral embolic protection device was associated with decreased prevalence of cognitive decline up to 1-week post-TAVI; (2) baseline cognitive impairment had a large association with post-TAVI cognitive improvement. In conclusion, cognitive decline and cognitive improvement are experienced by approximately 7% to 19% of patients after TAVI, respectively. Those with the lowest cognitive performance pre-TAVI appear to have the most to gain in terms of cognitive improvement post-TAVI. Identifying further predictive factors for cognitive decline and improvement post-TAVI will facilitate a personalized-medicine approach for cognitive care and prognosis.

Since its introduction in 2002, transcatheter aortic valve implantation (TAVI) has become standard care for severe symptomatic aortic stenosis in patients not suitable for surgical aortic valve replacement.^1^ TAVI has shown increased survival and reduced all-cause mortality compared with the surgical alternative, as well as long-term improvements in quality of life and functional capabilities.^1^, ^2^, ^3^ Previous systematic review and meta-analyses of cognitive outcomes after TAVI have shown, at a group-level, preservation (i.e., no significant decline or improvement) of cognitive function after TAVI.^4^^,^^5^ However, not all patient experiences align with this conclusion. Individual studies report a variety of cognitive outcomes (decline, no change, and improvement) experienced after TAVI.^6^, ^7^, ^8^, ^9^, ^10^ Knowledge of which patients are expected to cognitively decline and improve after TAVI is clinically relevant and important. This systematic review and meta-analysis synthesized existing literature to provide estimates of the prevalence of cognitive decline and improvement after TAVI. Additionally, an analysis of reported risk factors for cognitive decline and protective factors for cognitive improvement was conducted.

## Methods

This review was conducted in adherence with the Preferred Reporting Items for Systematic reviews and Meta-Analysis statement.^11^ Abstract, full-text screening and data extraction were conducted by 2 independent reviewers (E.S. Ghezzi and T.J. Ross). Conflicts were resolved through discussion. PsycINFO, Ovid Emcare, EMBASE, Pubmed, and Cochrane databases were searched using the search terms listed in the Supplement. Studies published from the time of the first TAVI procedure in 2002 until the search date (November 27, 2019) were included.

Inclusion criteria were peer-reviewed, full-text, English language studies investigating adult participants who underwent a TAVI procedure. Studies had to include both a pre- and postprocedure cognitive measure and report the number of participants who declined and/or improved post-TAVI relative to preprocedure cognitive function. Studies were excluded if only group-level changes to cognition were reported (i.e., average cognitive score pre- and post-TAVI), and if only mixed-samples were reported (e.g., TAVI and surgical aortic valve replacement). Case studies (n = 1), dissertations, book chapters, protocol papers, reviews, news articles, conference abstracts, letters to the editor, editorials and commentary publications were also excluded.

The quality of evidence and risk of bias were assessed using the Joanna Briggs Institute Critical Appraisal Checklist for Studies Reporting Prevalence Data^12^ (Supplement Table 1).

Data extracted included country, sample size, age, gender, definition of cognitive decline/improvement, frequency of cognitive decline/improvement at reported time-points post-TAVI, and reported predictive factors (pre-, intra-, and postoperative variables) for cognitive decline and improvement. Cognitive decline or improvement based on all cognitive assessments were included, from global cognitive function assessments (e.g., Montreal Cognitive Assessment [MoCA], Mini Mental State Examination [MMSE]) to individual tests (e.g., Trail Making Test, Digit Substitution Test, etc.). When 1 study reported 2 methods of determining cognitive decline within the same participants (e.g., decline in MoCA or MMSE), data from both methods were averaged within analyses.

Meta-analyses were completed using the Comprehensive Meta-Analysis Software (Version 3).^13^ Random-effects modelling was used due to considerable heterogeneity in study sample and design of included studies. Heterogeneity was assessed using the *I*^2^ statistic, with values of 25%, 50%, and 75% interpreted as small, moderate and large, respectively.^14^

Cognitive decline and improvement data were separated into 3 time-points post-TAVI: <1 month; ≥1 month and <6 months; and ≥6 months. Meta-analyses were then conducted to estimate prevalence of cognitive decline and cognitive improvement at each time-point. When 1 study reported 2 data points within 1 time period analysis (e.g., 2 and 7 days are both <1 month), both time points (measuring the same sample of participants) were averaged within analyses.

We further split these prevalence estimates by categorizing each study-reported definition of cognitive change (e.g., cognitive decline defined as ≥20% decrease in MoCA score) as relaxed or robust. Cognitive change definitions were categorized as relaxed if they did not attempt to account for measurement error or bias; simply counting any difference between baseline to post-TAVI test scores (≥1 point) as a relevant change, indicative of cognitive decline or improvement. Conversely, we defined robust cognitive change definitions as those which attempted to account for normal score variation in some way, either through a standard deviation cutoff,^6^^,^^9^ a reliable change index,^15^^,^^16^ or a score change (from baseline to post-TAVI) on a single cognitive test capturing general functioning (e.g., MoCA or MMSE) of ≥3 points.^17^, ^18^, ^19^, ^20^ Analyses to estimate the prevalence of cognitive decline or improvement based on cognitive change definition (robust and relaxed) were conducted at each time-point for which 2 or more papers reported cognitive change.

Risk factors were synthesized in the meta-analysis if they had been reported in at least 2 of the included studies. Ten independent risk factors met this criterion. The calculated outcomes are odds ratios (OR) for dichotomous risk factors and mean difference (MD) for continuous risk factors. Precision of the OR or MD was quantified by a 95% confidence interval [CI] for each analysis.

## Results

A total of 5,993 articles were identified, 4,632 after duplicates were removed. One thousand and sixty seven met criteria for full-text review and 15 of these were included for final review and meta-analyses (Supplement Figure 1). All 15 studies reported incidence of cognitive decline post-TAVI and 5 of these additionally reported the incidence of cognitive improvement post-TAVI (see Tables 1 and 2, respectively, for overview of these studies).

**Table 1:** Summary of included studies which report cognitive decline following transcatheter aortic valve implantation

Time-point	Study*	Country	Timing of assessment (post-TAVI) †	N at time-point	Age at time-point (years)**	Cognitive decline	Cognitive assessment	Cognitive decline definition	Robust/relaxed definition
<1 month	Fanning (2017)	Australia	3 days	31‡	82.4 (7.7)	9.7%	MoCA	Decrease ≥ 20%	Robust
	Fanning (2016)	Australia	3 days	40‡	81.7 (6.9)	5.0%	MoCA	Decrease ≥ 20%	Robust
	Ghanem (2013)	Germany	3 days	111	80 (6)	5.4%	RBANS	Decline > 1SD	Robust
	Gleason (2016)	USA	Discharge	79	-	2.5%	MMSE	Decrease > 4 points	Robust
	Haussig (2016)	Germany	2 days	72	-	61.1%	MoCA	Decrease ≥ 1 point	Relaxed
			7 days	72	-	48.6%	MoCA	Decrease ≥ 1 point	Relaxed
	Knipp (2013)	Germany	10.7 ± 4.9 days	22	-	18.2%	Mixed test battery 1	CCS ≤ –2	Robust
	Lansky (2015)	Europe and Israel	Predischarge	76	-	32.9%	MoCA	Worsened score	Relaxed
	Lansky (2016)	USA	Discharge	36	-	33.3%	MoCA	Worsened score	Relaxed
	Van Mieghem (2016)	Netherlands	5 ± 1 days	50	-	14.0%	MMSE	Worsened score	Relaxed
						16.0%	MoCA	Worsened score	Relaxed
	Zaleska Kockiecka (2018)	Poland	Discharge	38	-	42.1%	MMSE	Decrease ≥ 1 point	Relaxed
≥1 month, <6- months	Abawi (2018)	Netherlands	4 months	30	81 (6)	30.0%	IRMT	Worsened score	Relaxed
						23.3%	DRMT	Worsened score	Relaxed
						20.0%	RVIT	Worsened score	Relaxed
						30.0%	MMSE	Worsened score	Relaxed
						43.3%	TMT-A	Worsened score	Relaxed
						63.3%	TMT-B	Worsened score	Relaxed
						13.3%	CDT	Worsened score	Relaxed
	Altisent (2016)	Spain	79 ± 32 days	34	-	26.5%	GCD	RCI < –1	Robust
	Auffret (2016)	Canada	30 days	40	-	25.0%	Mixed test battery 2	RCI < –1.645 in ≥ 1 test	Robust
				51	80 [72–85]	7.8%	MoCA	RCI < –1.645	Robust
	Fanning (2017)	Australia	6 weeks	31‡	82.4 (7.7)	0%	MoCA	Decrease ≥ 20%	Robust
	Fanning (2016)	Australia	6 weeks	40‡	81.7 (6.9)	2.5%	MoCA	Decrease ≥ 20%	Robust
	Haussig (2016)	Germany	30 days	63	-	42.9%	MoCA	Decrease ≥ 1 point	Relaxed
	Knipp (2013)	Germany	115.6 ± 49.7 days	18	-	27.8%	Mixed test battery 1	CCS ≤ –2	Robust
	Lansky (2015)	Europe and Israel	30 days	64	-	31.3%	MoCA	Worsened score	Relaxed
	Lansky (2016)	USA	30 days	32	-	41.0%	MoCA	Worsened score	Relaxed
				28	-	32.1%	DSST	Worsened score	Relaxed
				29	-	20.7%	TMT-A (Err)	Increased errors	Relaxed
				32	-	37.3%	TMT-A (Time)	Increased time	Relaxed
				19	-	42.1%	TMT-B (Err)	Increased errors	Relaxed
				30	-	40.0%	TMT-B (Time)	Increased time	Relaxed
				32	-	53.1%	VF (Letter)	Worsened score	Relaxed
				32	-	56.3%	VF (Animal)	Worsened score	Relaxed
	Orvin (2014)	Israel	1 month	36	82.2 (4.2)	14.3%	MMSE	Worsened score	Relaxed
						11.4%	Cognistat test	Worsened score	Relaxed
						33.3%	Rouleau	Worsened score	Relaxed
≥6-months	Auffret (2016)	Canada	1 year	40	-	30.0%	Mixed test battery 2	RCI < –1.645 in ≥ 1 test	Robust
				51	80 [72–85]	11.8%	MoCA	RCI < –1.645	Robust
	Fanning (2016)	Australia	6 months	40‡	81.7 (6.9)	0%	MoCA	Decrease ≥ 20%	Robust
	Gleason (2016)	USA	1 year	62	-	8.1%	MMSE	Decrease >4 points	Robust
	Schoenenberger (2016)	Switzerland	6-9 months	229	83.4 (5.5)	12.7%	MMSE	Decrease ≥3 points	Robust

⁎ Included study reference list in Supplementary Materials.

⁎⁎ Age in years reported as mean (SD) or median [IQR].

† As reported within the study publication.

‡ Suspected sample overlap. CCS ≤–2 = Cognitive composite score ≤–2 (difference between number of tests with improvement [score difference ≥1SD] and decline [score difference ≤ -1SD]); CDT = Clock-drawing test; DRMT = Delayed recall memory test; DSST = Digit Symbol Substitution Test; GCD = Global cognitive dimension; IRMT = Immediate recall memory test; Mixed test battery 1 = Digit span subtest (Weschler Memory Scale-revised), wordlist subtest (Nümberg age inventory), Regensburg verbal fluency test, MMSE; Mixed test battery 2 = DSST, TMT-A, TMT-B, VF; MMSE = Mini Mental State Examination; MoCA = Montreal Cognitive Assessment; RBANS = Repeatable Battery for the Assessment of Neuropsychological Status; RCI = reliable change index; RVIT = Recognition of verbal information test; TAVI = transcatheter aortic valve implantation; TMT-A = Trail Making Test Part A; TMT-B = Trail Making Test Part B; VF = Verbal Fluency Tests.

**Table 2:** Summary of included studies which report cognitive improvement following transcatheter aortic valve implantation

Time-point	Study*	Country	Timing of assessment (post-TAVI) †	N at time-point	Age at time-point (years)‡	Cognitive decline	Cognitive assessment	Cognitive improvement definition	Robust/relaxed definition
<1-month	Lansky (2016)	USA	Discharge	36	-	50.0%	MoCA	Improved score	Relaxed
≥1-month, <6-month	Altisent (2016)	Spain	79 ± 32 days	34	-	20.6%	GCD	RCI > 1	Robust
	Auffret (2016)	Canada	30 days	40	-	40.0%	Mixed test battery	RCI > 1.645 in ≥ 1 test	Robust
				51	80 [72–85]	5.9%	MoCA	RCI > 1.645	Robust
	Lansky (2016)	USA	30 days	32	-	38.0%	MoCA	Improved score	Relaxed
				28	-	60.7%	DSST	Improved score	Relaxed
				29	-	10.3%	TMT-A (Err)	Decreased errors	Relaxed
				32	-	53.1%	TMT-A (Time)	Decreased time	Relaxed
				19	-	31.6%	TMT-B (Err)	Decreased errors	Relaxed
				30	-	33.3%	TMT-B (Time)	Decreased time	Relaxed
				32	-	31.2%	VF (Letter)	Improved score	Relaxed
				32	-	40.6%	VF (Animal)	Improved score	Relaxed
	Orvin (2014)	Israel	1 month	36	82.2 (4.2)	55.6%	MMSE	Improved score	Relaxed
≥6-month	Auffret (2016)	Canada	1 year	40	-	30.0%	Mixed test battery	RCI > 1.645 in ≥ 1 test	Robust
				51	80 [72–85]	7.8%	MoCA	RCI > 1.645	Robust
	Schoenenberger (2016)	Switzerland	6-9 months	229	83.4 (5.5)	10.5%	MMSE	Decrease ≥ 3 points	Robust

⁎ Included study reference list in Supplementary Materials.

† As reported within the study publication.

‡ Age in years reported as mean (SD) or median [IQR]. DSST = Digit Symbol Substitution Test; GCD = Global cognitive dimension; Mixed test battery = DSST, TMT-A, TMT-B, VF; MMSE = Mini Mental State Examination; MoCA = Montreal Cognitive Assessment; RCI = reliable change index; TAVI = transcatheter aortic valve implantation; TMT-A = Trail Making Test Part A; TMT-B = Trail Making Test Part B; VF = Verbal Fluency Tests.

**Figure 1:**
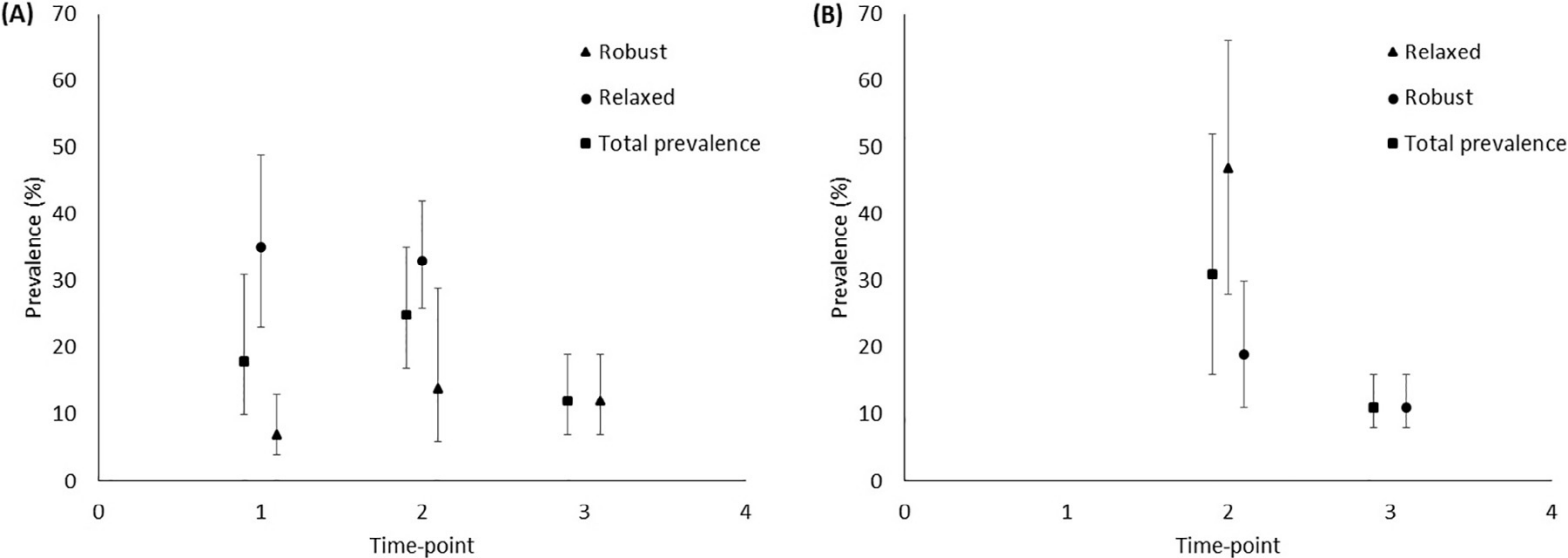
Prevalence of *(A)* cognitive decline and *(B)* cognitive improvement based on relaxed, robust, and both (total prevalence) definitions of cognitive change at time-points after transcatheter aortic valve implantation. Time-point 1 ≤1 month; Time-point 2 ≥1 month and <6 months; Time-point 3 ≥6 months. Error bars represent 95% CI.

The pooled prevalence of cognitive decline (regardless of relaxed/robust definition) was 18% (95% CI 10% to 31%, *I^2^* = 88.77) up to 1 month, 25% (95% CI 17% to 35%, *I^2^* = 65.11) from 1 to 6 months, and 12% (95% CI 7% to 19%, *I^2^* = 48.09) 6 months and beyond after TAVI (Figure 1).

The pooled prevalence of cognitive improvement was 31% (95% CI 16% to 52%, *I^2^* = 77.56) from 1 to 6 months (regardless of relaxed/robust definition), and 11% (95% CI 8% to 16%, *I^2^* = 0) 6 months and beyond (based on robust definition) after TAVI (Figure 1). The prevalence of cognitive improvement up to 1 month after TAVI was not analyzed as only 1 study^21^ reported relevant data (50% cognitive improvement at discharge).

The pooled prevalence of cognitive decline up to 1-month post-TAVI when using a relaxed cognitive change definition was 35% (95% CI 23% to 49%, *I^2^* = 79.66) versus 7% (95% CI 4% to 13%, *I^2^* = 43.28) when a robust cognitive change definition was employed (Figure 1). The pooled prevalence of cognitive decline 1 to 6 months post-TAVI when using a relaxed cognitive change definition was 33% (95% CI 26% to 42%, *I^2^* = 37.42) and 14% (95% CI 6% to 29%, *I^2^* = 63.54) when a robust cognitive change definition was utilized (Figure 1). The pooled prevalence of cognitive improvement 1 to 6 months post-TAVI when using a relaxed cognitive change definition was 47% (95% CI 28% to 66%, *I^2^* = 55.76) and 19% (95% CI 11% to 30%, *I^2^* = 0) when a robust cognitive change definition was used (Figure 1).

Forest plots and heterogeneity statistics for prevalence analyses can be seen in Supplement Table 2 and Supplement Table 3.

Ten risk factors for the development of cognitive decline after TAVI were separately analyzed (see Table 3 and Supplement Table 4). These comprised 8 preprocedural factors (age, atrial fibrillation, body mass index, diabetes, gender, hypertension, baseline cognitive impairment, and stroke/TIA), 1 intraprocedural factor (cerebral protection device) and 1 postprocedural factor (stroke). Sufficient data were available to split the risk factor analysis for cerebral protection device into 2 time-points; up to 1-week/discharge, and 1-month post-TAVI; all other data were merged across time-points.

**Table 3:** Meta-analyses on pre-, intra-, and postprocedural variables for the development of cognitive decline and cognitive improvement following transcatheter aortic valve implantation

Variable	Studies	Time-point of decline	N	OR or MD* (95% CI)	p value	Heterogeneity
Factors associated with cognitive decline						
Preprocedural						
Age	Ghanem, 2013; Schoenenberger 2016	All	340	1.44* (–2.05–4.92)	0.420	*I*^2^ = 48.36, df = 1, p = 0.164
AF	Ghanem, 2013; Lansky, 2015; Schoenenberger, 2016	All	410	1.57 (0.84–2.92)	0.156	*I*^2^ = 0, df = 2, p = 0 .374
BMI	Ghanem, 2013; Schoenenberger, 2016	All	340	0.73* (–1.21–2.66)	0.462	*I*^2^ = 0, df = 1, p = 0.635
Diabetes	Ghanem, 2013; Schoenenberger, 2016	All	340	0.76 (0.32–1.82)	0.535	*I^2^* = 0*,* df =1, p = 0.560
Gender	Ghanem, 2013; Schoenenberger, 2016	All	340	1.27 (0.62–2.59)	0.507	*I*^2^ = 0, df = 1, p = 0.766
Hypertension	Ghanem, 2013; Schoenenberger, 2016	All	340	1.33 (0.16–10.84)	0.787	*I*^2^ = 26.88, df = 1, p = 0.242
Baseline CI	Ghanem, 2013; Schoenenberger, 2016	All	340	0.79 (0.26–2.36)	0.668	*I*^2^ = 9.24, df = 1, p = 0.294
Stroke/TIA	Ghanem, 2013; Schoenenberger, 2016	All	340	1.35 (0.48–3.81)	0.568	*I*^2^ = 0, df = 1, p = 0.328
Intraprocedural						
Cerebral protection device	Haussig, 2016; Lansky, 2015; VanMieghem, 2016	Up to 1-week/discharge	198	0.47 (0.25–0.90)	0.022	*I*^2^ = 0, df = 2, p = 0.608
Cerebral protection device	Haussig, 2016; Lansky, 2015	1-month	127	1.00 (0.48–2.07)	0.993	*I*^2^ = 0, df = 1, p = 0.336
Postprocedural						
Stroke	Ghanem, 2013⁎; Gleason, 2016†; Lansky, 2016‡; Schoenenberger, 2016§	All	325	0.54 (0.11–2.71)	0.452	*I*^2^ = 22.02, df = 2, p = 0.277
Factors associated with cognitive improvement						
Baseline CI	Auffret, 2016; Schoenenberger, 2016	All	280	14.54 (5.79–36.52)	<0.001	*I*^2^ = 0*,* df = 1, p = 0.357

⁎ No patients had a post-procedure stroke.

† Any stroke or TIA.

‡ Stroke based on VARC-2 criteria.

§ Stroke/MI within 30 days, based on VARC criteria. AF =atrial fibrillation; BMI = body mass index; CI = cognitive impairment; MD = mean difference; OR = odds ratio; TIA = transient ischemic attack.

Using a cerebral protection device was significantly associated with lower prevalence of cognitive decline up to 1-week post-TAVI (OR 0.47, 95% CI 0.25 to 0.90, p = 0.022). However, at 1-month post-TAVI, using a cerebral protection device was not significantly associated with prevalence of cognitive decline (OR 1.00, 95% CI 0.48 to 2.07, p = 0.993). No other factors were significantly associated with cognitive decline.

Protective factors for cognition after TAVI were not commonly reported. The only factor that was reported by more than 1 paper was baseline cognitive impairment. As such, baseline cognitive impairment was the sole variable meta-analyzed for the development of cognitive improvement (see Table 3 and Supplement Table 5), with the association found to be significant (OR 14.5, 95% CI 5.8 to 36.5, p <0.001).

## Discussion

This meta-analysis synthesized available data representing cognitive decline and improvement in TAVI patients over time. Studies using relaxed definitions of cognitive change (which do not account for measurement error and practice effects) likely overestimated the incidence and clinical significance of both cognitive decline and improvement. To account for normal individual variation in cognitive tests over time, we suggest current best estimates for prevalence of cognitive decline and improvement after TAVI come from robust cognitive change definitions. As such, we report that best estimates of prevalence of cognitive decline up to 1-month, 1 to 6-months, and ≥6-months post-TAVI are 7%, 14%, and 12%, respectively (robust estimates). For cognitive improvement, the prevalence from 1 to 6 months, and ≥6 months after TAVI is estimated at 19% and 11%, respectively (robust estimates).

Caution should be employed when interpreting these results as pooled estimates are not directly comparable across time-points and between cognitive decline and improvement because they represent different individuals. Results do seem to indicate that cognitive decline and improvement are both commonly occurring outcomes after TAVI procedures, with incidence of cognitive decline and improvement estimated to be between 7% and 19% in the 6 months after TAVI.

It appears as though equivalent numbers (given CIs overlap) of TAVI patients experience cognitive decline and cognitive improvement, which has given rise to the null findings using a whole-group-analysis approach previously.^4^^,^^5^ However, it is critical that we try to understand the heterogeneity in this effect, including those at high risk of cognitive decline and improvement post-TAVI. If we can understand the predictive factors for cognitive decline and improvement, we can provide personalized care and improve prognosis.

We found minimal consistent reporting of risk factors for cognitive decline after TAVI in the literature. As such, we were only able to meta-analyze the effects of 10 factors on their contribution to cognitive decline. Of the analyzed factors, only use of a cerebral protection device was found to have a statistically significant association with lower prevalence of cognitive decline up to 1-week post-TAVI, which was no longer significant at 1 month. As patients in these studies either used a protection device or did not, patient characteristics may have influenced these associations.

Cognitive improvement is a relatively common outcome after TAVI, occurring in an estimated 19% of patients up to 6-months postprocedure. Baseline cognitive impairment was the only factor able to be analyzed and was found to have a large association with post-TAVI cognitive improvement. Those with the lowest cognitive function pre-TAVI have the most to gain post-TAVI; but it also may be the case that ceiling effects existed within some of the tests used in contributing studies (partly driving this effect). With a large proportion of the generally older population^22^ who underwent TAVI presenting with preprocedure cognitive impairment (approx. 30%),^23^, ^24^, ^25^ these results suggest there is great potential for cognitive improvement after TAVI in these patients.

Limited reporting of risk factors meant the effect of important variables on cognitive outcomes after TAVI were unable to be analyzed. This general lack of information on predictors of cognitive decline post-TAVI is somewhat surprising considering a recent meta-analysis which identified 8 risk factors for delirium after TAVI.^26^ Delirium is another incident cognitive impairment, but one which is temporary.^27^ As such, the impact of risk factors identified for delirium, including acute kidney injury, transapical approach, and carotid artery disease,^26^ on post-TAVI cognitive decline should be measured in future studies. An investigation of risk factors previously identified for cognitive decline after heart surgery, particularly depression,^28^ in relation to post-TAVI cognitive outcomes would also be useful. Such information will allow a more comprehensive analysis of the predictors of cognitive decline and improvement after TAVI.

It should be noted that 3 of the reported risk factors for delirium after TAVI (hypertension, atrial fibrillation, previous stroke)^26^ were also assessed in the current study and were not associated with post-TAVI cognitive decline. Additionally, whereas Tilley et al^26^ showed pre-TAVI cognitive impairment as a risk factor for delirium, in the current study baseline cognitive impairment was associated with cognitive improvement. To better inform clinical practice, further research is needed to compare predictive factors for cognitive decline and delirium after TAVI. These contrary findings may be due to smaller sample sizes here, and the assessment of subgroups rather than whole-group averages.

Although this meta-analysis was the first to synthesize available literature and report the prevalence of cognitive decline and improvement after TAVI, it was not without limitations. Only 15 studies met inclusion criteria, resulting in smaller sample sizes within analyses. Moderate to large heterogeneity was found in prevalence analyses at all time-points which included both relaxed and robust definitions of cognitive change. Splitting analyses by these definitions increased homogeneity. The reliable change index method, which corrects for practice and ageing effects, has been shown to perform well in signifying change associated with a change in diagnostic status.^29^ We suggest future studies should employ a reliable change index method for classifying significant cognitive change, as it considers individual change in comparison to the group but does not utilize arbitrary cutoffs. Furthermore, the scarcity of risk factors reported in included studies made a comprehensive analysis of predictors of cognitive change after TAVI unable to be completed. More research is required in this area which reports potential predictors of cognitive change post-TAVI (e.g., depression, acute kidney injury, and transapical approach).

TAVI has quickly gained traction as the standard-of-care in the treatment of severe, symptomatic aortic stenosis for individuals ineligible for the surgical alternative. Notably, the current review highlighted that cognitive decline, an important but often overlooked post-TAVI outcome, is just as frequent an occurrence as cognitive improvement (ranging from 7% to 19%). The current paper also revealed that most individuals, approximately 81% to 93%, do not experience any long-term cognitive change post-TAVI. Assessment of these subgroups will enable a personalized medicine approach in TAVI, particularly the development and targeting of neuroprotective strategies in those at high risk of cognitive decline.

## Declaration of Interests

The authors declare that they have no known competing financial interests or personal relationships that could have appeared to influence the work reported in this paper.

## Disclosures

The authors report no relationships that could be construed as a conflict of interest.

## Appendix Supplementary materials

Image, application 1

## Footnotes

Supplementary material associated with this article can be found in the online version at https://doi.org/10.1016/j.amjcard.2020.04.023.

## References

[1] Leon MB, Smith CR, Mack M, Miller DC, Moses JW, Svensson LG, Tuzcu EM, Webb JG, Fontana GP, Makkar RR, Brown DL, Block PC, Guyton RA, Pichard AD, Bavaria JE, Herrmann HC, Douglas PS, Petersen JL, Akin JJ, Anderson WN, Wang D, Pocock S (2010). Transcatheter aortic-valve implantation for aortic stenosis in patients who cannot undergo surgery. N Engl J Med.

[2] Adams DH, Popma JJ, Reardon MJ, Yakubov SJ, Coselli JS, Deeb GM, Gleason TG, Buchbinder M, Hermiller J, Kleiman NS, Chetcuti S, Heiser J, Merhi W, Zorn G, Tadros P, Robinson N, Petrossian G, Hughes C, Harrison K, Conte J, Maini B, Mumtaz M, Chenoweth S, Oh JK (2014). Transcatheter aortic-valve replacement with a self-expanding prosthesis. N Engl J Med.

[3] Georgiadou P, Kontodima P, Sbarouni E, Karavolias GK, Smirli A, Xanthos T, Troupis T, Khouri M, Papadimitriou L, Voudris V (2011). Long-term quality of life improvement after transcatheter aortic valve implantation. Am Heart J.

[4] Khan MM, Herrmann N, Gallagher D, Gandell D, Fremes SE, Wijeysundera HC, Radhakrishnan S, Sun YR, Lanctot KL (2018). Cognitive outcomes after transcatheter aortic valve implantation: a metaanalysis. J Am Geriatr Soc.

[5] Lai KSP, Herrmann N, Saleem M, Lanctôt KL (2015). Cognitive outcomes following transcatheter aortic valve implantation: a systematic review. Cardiovasc Psychiatry Neurol.

[6] Ghanem A, Kocurek J, Sinning J-M, Wagner M, Becker BV, Vogel M, Schröder T, Wolfsgruber S, Vasa-Nicotera M, Hammerstingl C, Schwab JO, Thomas D, Werner N, Grube E, Nickenig G, Muller A (2013). Cognitive trajectory after transcatheter aortic valve implantation. Circ Cardiovasc Interv.

[7] Orvin K, Dvir D, Weiss A, Assali A, Vaknin-Assa H, Shapira Y, Gazit O, Sagie A, Kornowski R (2014). Comprehensive prospective cognitive and physical function assessment in elderly patients undergoing transcatheter aortic valve implantation. Cardiology.

[8] Kahlert P, Al-Rashid F, Döttger P, Mori K, Plicht B, Wendt D, Bergmann L, Kottenberg E, Schlamann M, Mummel P, Holle D, Thielmann M, Jakob HG, Konorza T, Heusch G, Erbel R, Eggebrecht H (2012). Cerebral embolization during transcatheter aortic valve implantation: a transcranial Doppler study. Circulation.

[9] Knipp SC, Kahlert P, Jokisch D, Schlamann M, Wendt D, Weimar C, Jakob H, Thielmann M (2013). Cognitive function after transapical aortic valve implantation: a single-centre study with 3-month follow-up. Interact Cardiovasc Thorac Surg.

[10] Rodés-Cabau J, Dumont E, Boone RH, Larose E, Bagur R, Gurvitch R, Bédard F, Doyle D, De Larochellière R, Jayasuria C, Villeneuve J, Marrero A, Cote M, Pibarot P, Webb JG (2011). Cerebral embolism following transcatheter aortic valve implantation: comparison of transfemoral and transapical approaches. J Am Coll Cardiol.

[11] Liberati A, Altman DG, Tetzlaff J, Mulrow C, Gøtzsche PC, Ioannidis JPA, Clarke M, Devereaux PJ, Kleijnen J, Moher D (2009). The PRISMA statement for reporting systematic reviews and meta-analyses of studies that evaluate health care interventions: explanation and elaboration. Ann Intern Med.

[12] Joanna Briggs Institute (2011). Joanna Briggs Institute Critical Appraisal Checklist for Studies Reporting Prevalence Data.

[13] Borenstein M, Hedges L, Higgins J, Rothstein H. Comprehensive Meta-Analysis Version 3, 2013, Englewood, NJ: Biostat.

[14] Higgins JP, Thompson SG, Deeks JJ, Altman DG (2003). Measuring inconsistency in meta-analyses. BMJ.

[15] Altisent OA-J, Ferreira-Gonzalez I, Marsal JR, Ribera A, Auger C, Ortega G, Cascant P, Urena M, Del Blanco BG, Serra V, Sureda C, Igual A, Rovira A, Gonzalez-Alujas MT, Gonzalez A, Puri R, Cuellar H, Tornos P, Rodes-Cabau J, Garcia-Dorado D (2016). Neurological damage after transcatheter aortic valve implantation compared with surgical aortic valve replacement in intermediate risk patients. Clin Res Cardiol.

[16] Auffret V, Campelo-Parada F, Regueiro A, Del Trigo M, Chiche O, Chamandi C, Allende R, Cordoba-Soriano JG, Paradis JM, De Larochellière R, Doyle D, Dumont E, Mohammadi S, Cote M, Marrero A, Puri R, Rodes-Cabau J (2016). Serial changes in cognitive function following transcatheter aortic valve replacement. J Am Coll Cardiol.

[17] Fanning JP, Walters DL, Wesley AJ, Anstey C, Huth S, Bellapart J, Collard C, Rapchuk IL, Natani S, Savage M, Fraser JF (2017). Intraoperative cerebral perfusion disturbances during transcatheter aortic valve replacement. Ann Thorac Surg.

[18] Fanning JP, Wesley AJ, Walters DL, Eeles EM, Barnett AG, Platts DG, Clarke AJ, Wong AA, Strugnell WE, O'Sullivan C, Tronstad O, Fraser JF (2016). Neurological injury in intermediate‐risk transcatheter aortic valve implantation. J Am Heart Assoc.

[19] Gleason TG, Schindler JT, Adams DH, Reardon MJ, Kleiman NS, Caplan LR, Conte JV, Deeb GM, Hughes GC, Chenoweth S, Popma JJ (2016). The risk and extent of neurologic events are equivalent for high-risk patients treated with transcatheter or surgical aortic valve replacement. J Thorac Cardiovasc Surg.

[20] Schoenenberger AW, Zuber C, Moser A, Zwahlen M, Wenaweser P, Windecker S, Carrel T, Stuck AE, Stortecky S (2016). Evolution of cognitive function after transcatheter aortic valve implantation. Circ Cardiovasc Interv.

[21] Lansky AJ, Brown D, Pena C, Pietras CG, Parise H, Ng VG, Meller S, Abrams KJ, Cleman M, Margolis P, Petrossian G, Brickman AM, Voros S, Moses J, Forrest JK (2016). Neurologic complications of unprotected transcatheter aortic valve implantation (from the Neuro-TAVI Trial). Am J Cardiol.

[22] Alkhalil A, Golbari S, Song D, Lamba H, Fares A, Alaiti A, Deo S, Attizzani GF, Ibrahim H, Ruiz CE (2018). In‐hospital outcomes of transcatheter versus surgical aortic valve replacement in end stage renal disease. Catheter Cardiovasc Interv.

[23] Schoenenberger AW, Stortecky S, Neumann S, Moser A, Jüni P, Carrel T, Huber C, Gandon M, Bischoff S, Schoenenberger C-M, Stuck AE, Windecker S, Wenaweser P (2013). Predictors of functional decline in elderly patients undergoing transcatheter aortic valve implantation (TAVI). Eur Heart J.

[24] Stortecky S, Schoenenberger AW, Moser A, Kalesan B, Jüni P, Carrel T, Bischoff S, Schoenenberger C-M, Stuck AE, Windecker S, Wenaweser P (2012). Evaluation of multidimensional geriatric assessment as a predictor of mortality and cardiovascular events after transcatheter aortic valve implantation. JACC Cardiovasc Interv.

[25] Tse L, Bowering JB, Schwarz SK, Moore RL, Burns KD, Barr AM (2015). Postoperative delirium following transcatheter aortic valve implantation: a historical cohort study. Can J Anesth Can d'anesthésie.

[26] Tilley E, Psaltis PJ, Loetscher T, Davis DH, Harrison SL, Kim S, Keage HAD (2018). Meta-analysis of prevalence and risk factors for delirium after transcatheter aortic valve implantation. Am J Cardiol.

[27] Fong TG, Davis D, Growdon ME, Albuquerque A, Inouye SK (2015). The interface between delirium and dementia in elderly adults. Lancet Neurol.

[28] Greaves D, Psaltis PJ, Davis D, Ross T, Ghezzi E, Lampit A, Smith A, Keage HAD. Risk factors for delirium and cognitive decline following coronary artery bypass grafting surgery: a systematic review and meta-analysis, Under review. 10.1161/JAHA.120.017275 PMC7763731 33164631

[29] Frerichs RJ, Tuokko HA (2005). A comparison of methods for measuring cognitive change in older adults. Arch Clin Neuropsychol.

