# Supplementary material for: Meta-Analysis of Prevalence and Risk Factors for Cognitive Decline and Improvement After Transcatheter Aortic Valve Implantation
Source: Am J Cardiol. 2020 Jul 15;127:105–12. doi: 10.1016/j.amjcard.2020.04.023 (PMC8376655; doi:10.1016/j.amjcard.2020.04.023)
Supplement: Supplementary file 1 [file mmc1.docx]

**Supplemental Methods**

**Search Terms by database:**

**PsycINFO, Ovid Emcare & Embase**

(TAVR.mp OR TAVI.mp OR transcatheter aortic valve.mp OR aortic valve replacement.mp OR transcatheter.mp OR valve replacement.mp) AND (exp cognition OR cogn*.mp OR exp delirium OR delirium.mp OR exp dementia OR dementia.mp)

**Pubmed:**

(TAVI[tw] OR TAVR[tw] OR transcatheter aortic valve[tw] OR transcatheter[tw] or valve replacement[tw] OR "transcatheter aortic valve replacement"[mh]) AND (cogn*[tw] OR "cognition"[mh] OR "cognition disorders"[mh] OR delirium[tw] OR "delirium"[mh] OR dementia[tw] OR "dementia"[mh])

**Cochrane:**

(TAVR):ti,ab,kw OR (TAVI):ti,ab,kw OR ("transcatheter aortic valve"):ti,ab,kw OR ("aortic valve replacement"):ti,ab,kw OR (transcatheter):ti,ab,kw OR ("valve replacement"):ti,ab,kw OR MeSH descriptor: [Transcatheter Aortic Valve Replacement] explode all trees AND MeSH descriptor: [Cognition] explode all trees OR (cogn*):ti,ab,kw OR MeSH descriptor: [Delirium] explode all trees OR (delirium):ti,ab,kw OR MeSH descriptor: [Dementia] explode all trees OR (Dementia):ti,ab,kw

**Supplemental Tables**

Table 1: Critical appraisal scores for case studies meeting eligibility for inclusion for the review using the Joanna Briggs Institute Critical Appraisal Checklist for Prevalence Studies ^1^

| ***Author/year*** | **Abawi et al. 2017 ^2^** | **Altisent et al. 2016 ^3^** | **Auffret et al. 2016 ^4^** | **Fanning et al. 2016 ^5^** | **Fanning et al. 2017 ^6^** | **Ghanem et al. 2013 ^7^** | **Gleason et al. 2016 ^8^** | **Haussig et al. 2016 ^9^** | **Knipp et al. 2013 ^10^** | **Lansky et al. 2015 ^11^** | **Lansky et al. 2016 ^12^** | **Orvin et al. 2014 ^13^** | **Schoenenberger et al. 2016 ^14^** | **Van Mieghem et al. 2016 ^15^** | **Zaleska-Kockiecka, 2018 ^16^** |
| --- | --- | --- | --- | --- | --- | --- | --- | --- | --- | --- | --- | --- | --- | --- | --- |
| 1. Was the sample frame appropriate to address the target population? | *Yes* | *Yes* | *Unclear* | *Yes* | *Unclear* | *Yes* | *Yes* | *Yes* | *No* | *Yes* | *Yes* | *Yes* | *Yes* | *Yes* | *Yes* |
| 2. Were study participants sampled in an appropriate way? | *Yes* | *Yes* | *Unclear* | *Yes* | *Yes* | *Yes* | *Yes* | *Yes* | *Yes* | *Unclear* | *Yes* | *Unclear* | *Yes* | *Yes* | *Yes* |
| 3. Was the sample size adequate?* | *No* | *No* | *No* | *No* | *No* | *No* | *No* | *No* | *No* | *No* | *No* | *No* | *No* | *No* | *No* |
| 4. Were the study subjects and the setting described in detail? | *Yes* | *Yes* | *Yes* | *Yes* | *Yes* | *Yes* | *Yes* | *Yes* | *Yes* | *Yes* | *Yes* | *Yes* | *Yes* | *Yes* | *Yes* |
| 5. Was the data analysis conducted with sufficient coverage of the identified sample? | *Yes* | *No* | *Yes* | *Unclear* | *Yes* | *Yes* | *Unclear* | *No* | *No* | *No* | *No* | *Yes* | *Yes* | *No* | *No* |
| 6. Were valid methods used for the identification of the condition? | *No* | *Yes* | *Yes* | *Yes* | *Yes* | *Yes* | *Yes* | *No* | *Yes* | *No* | *No* | *No* | *Yes* | *No* | *No* |
| 7. Was the condition measured in a standard, reliable way for all participants? | *Unclear* | *Yes* | *Yes* | *Unclear* | *Unclear* | *Yes* | *Yes* | *Yes* | *Unclear* | *Yes* | *Yes* | *Yes* | *Yes* | *Yes* | *Yes* |
| 8. Was there appropriate statistical analysis? | *No* | *Yes* | *No* | *No* | *No* | *Unclear* | *No* | *No* | *No* | *No* | *Yes* | *No* | *No* | *No* | *No* |
| 9. Was the response rate adequate, and if not, was the low response rate managed appropriately? | *Yes* | *No* | *Yes* | *Unclear* | *Yes* | *Unclear* | *No* | *Unclear* | *Yes* | *Unclear* | *Unclear* | *Yes* | *Yes* | *Unclear* | *No* |
| **Total score:** | **5** | **6** | **5** | **4** | **5** | **6** | **5** | **4** | **4** | **3** | **5** | **5** | **7** | **4** | **4** |

* The following formula was used to determine whether or not the included studies utilized an adequate sample size; $n= \frac{Z^{2}P(1-P)}{d^{2}}$ where Z is the Z statistic for a level of confidence, P is the expected prevalence or proportion, and d is the precision. Where estimated prevalence is unknown (as a meta-analysis on the prevalence of cognitive decline following TAVI has not been conducted previously), conservative assumptions are preferable (up to 50%) ^17-19^. Thus, assuming 5% precision (*d* = 0.05) with 95% CI (*Z* = 1.96) and a conservative estimate of prevalence (*P* = 0.5), an adequately powered study would have a sample size of at least 385 participants.

Table 2: Forest plots and heterogeneity for pooled prevalence of cognitive decline following transcatheter aortic valve implantation.

| **Time-point after TAVI** | **Cognitive change definition** | **Forest Plot** | **Heterogeneity** |
| --- | --- | --- | --- |
| <1-month | Combined (robust and relaxed) |  | *I^2^* = 88.77, df=9, p<.001 |
| ≥1-month and <6-months | Combined (robust and relaxed) |  | *I^2^* = 65.11, df=9, p=.002 |
| ≥6-months | Combined (robust and relaxed) |  | *I^2^* = 48.09, df=3, p=.123 |
| <1-month | Relaxed |  | *I^2^* = 79.66, df=4, p=.001 |
| <1-month | Robust |  | *I^2^* = 43.28, df=4, p=.133 |
| ≥1-month and <6-months | Relaxed |  | *I^2^* = 37.42, df=4, p=.172 |
| ≥1-month and <6-months | Robust |  | *I^2^* = 63.54, df=4, p=.027 |

TAVI = transcatheter aortic valve implantation. Combined comparisons are instances where authors reported separate cognitive decline prevalence results for multiple individual tests. In these cases, prevalence of cognitive improvement/decline on the tests in the study was pooled.

Table 3: Forest plots and heterogeneity for pooled prevalence of cognitive improvement following transcatheter aortic valve implantation.

| **Time-point after TAVI** | **Cognitive change definition** | **Forest Plot** | **Heterogeneity** |
| --- | --- | --- | --- |
| ≥1-month and <6-months | Combined (robust and relaxed) |  | *I^2^* = 77.56, df=3, p=.004 |
| ≥6-months | Combined (robust and relaxed) |  | *I^2^* = 0, df=1, p=.320 |
| ≥1-month and <6-months | Relaxed |  | *I^2^* = 55.76, df=1, p=.133 |
| ≥1-month and <6-months | Robust |  | *I^2^* = 0, df=1, p=.709 |

TAVI = transcatheter aortic valve implantation. Combined comparisons are instances where authors reported separate cognitive decline prevalence results for multiple individual tests. In these cases, prevalence of cognitive improvement/decline on the tests in the study was pooled.

Table 4: Forest plots and heterogeneity for pre- and post-procedural variables for the development of cognitive impairment following transcatheter aortic valve implantation.

| **Variable** | **Forest Plot** | **Heterogeneity** |
| --- | --- | --- |
| **Pre-procedural** |  |  |
| Age* |  | *I^2^ =* 48.36*,* df=1, p=.164 |
| AF |  | *I^2^ =* 0*,* df=2, p=.374 |
| BMI* |  | *I^2^ =* 0, df=1, p=.635 |
| Diabetes |  | *I^2^ =* 0, df=1, p=.560 |
| Gender |  | *I^2^ =* 0, df=1, p=.766 |
| Hypertension |  | *I^2^ =* 26.88, df=1, p=.242 |
| Baseline CI |  | *I^2^ =* 9.24, df=1, p=.294 |
| Prior Stroke/TIA |  | *I^2^ =* 0, df=1, p=.328 |
| **Intra-procedural** |  |  |
| Cerebral protection device (Cognitive decline up to 1 week/discharge) |  | *I^2^ =* 0, df=2, p=.608 |
| Cerebral protection device (Cognitive decline 1-month post-TAVI) |  | *I^2^* = 0*,* df=1, p=.336 |
| **Post-procedural** |  |  |
| Post-procedural Stroke |  | *I^2^ =*22.02, df=2, p= .277 |

* Reported as mean difference. AF= atrial fibrillation, BMI= Body Mass Index, CI= cognitive impairment, TAVI= transcatheter aortic valve implantation, and TIA= transient ischemic attack.

**Table 5: Forest plots and heterogeneity for pre- and post-procedural variables for the development of cognitive improvement following transcatheter aortic valve implantation.**

| **Variable** | **Forest Plot** | **Heterogeneity** |
| --- | --- | --- |
| Baseline CI |  | *I^2^* = 0, df=1, p=.357 |

CI= cognitive impairment.

**Supplemental Figures**

Records identified through database searching
(n = 5990)

## Screening

## Included

## Eligibility

## Identification

Records after duplicates removed
(n = 4675)

Records screened
(n = 4675)

Records excluded
(n = 3604)

Full-text articles assessed for eligibility
(n = 1071)

Full-text articles excluded, with reasons (n = 1056)

Not in English (n = 26)

Abstract only (n = 389)

Comment/letter (n = 226)

Dissertation (n = 2)

Review (n = 68)

Case study (n = 28)

Not a quantitative study (n = 24)

Duplicate sample (n = 44)

No TAVI (n = 134)

Mixed sample (n = 1)

Both pre- and post-TAVI cognition not reported (n = 94)

Individual change not reported (n = 20)

Studies included in qualitative synthesis
(n = 15)

Studies included in quantitative synthesis (meta-analysis)
(n = 15)

**Figure 1.**

***PRISMA flow diagram. TAVI = transcatheter aortic valve implantation.***

**Supplemental References**

**1.** Munn Z, Moola S, Lisy K, Riitano D, Tufanaru C. Methodological guidance for systematic reviews of observational epidemiological studies reporting prevalence and cumulative incidence data. *Int J Evid Based Healthc* 2015;13:147-153.

**2.** Abawi M, de Vries R, Stella PR, Agostoni P, Boelens DHM, van Jaarsveld RC, van Dongen CS, Doevendans PAFM, Emmelot-Vonk MH. Evaluation of cognitive function following transcatheter aortic valve replacement. *Heart Lung Circ* 2018;27:1454–1461.

**3.** Altisent OA-J, Ferreira-Gonzalez I, Marsal JR, Ribera A, Auger C, Ortega G, Cascant P, Urena M, Del Blanco BG, Serra V, Sureda C, Igual A, Rovira A, Gonzalez-Alujas MT, Gonzalez A, Puri R, Cuellar H, Tornos P, Rodes-Cabau J, Garcia-Dorado D. Neurological damage after transcatheter aortic valve implantation compared with surgical aortic valve replacement in intermediate risk patients. *Clin Res Cardiol* 2016;105:508–517.

**4.** Auffret V, Campelo-Parada F, Regueiro A, Del Trigo M, Chiche O, Chamandi C, Allende R, Cordoba-Soriano JG, Paradis JM, De Larochelliere R, Doyle D, Dumont E, Mohammadi S, Cote M, Marrero A, Puri R, Rodes-Cabau J. Serial changes in cognitive function following transcatheter aortic valve replacement. *J Am Coll Cardiol* 2016;68:2129-2141.

**5.** Fanning JP, Wesley AJ, Walters DL, Eeles EM, Barnett AG, Platts DG, Clarke AJ, Wong AA, Strugnell WE, O’Sullivan C, Tronstad O, Fraser JF. Neurological injury in intermediate‐risk transcatheter aortic valve implantation. *J Am Heart Assoc* 2016;5:e004203.

**6.** Fanning JP, Walters DL, Wesley AJ, Anstey C, Huth S, Bellapart J, Collard C, Rapchuk IL, Natani S, Savage M, Fraser JF. Intraoperative cerebral perfusion disturbances during transcatheter aortic valve replacement. *Ann Thorac Surg* 2017;104:1564–1568.

**7.** Ghanem A, Kocurek J, Sinning J-M, Wagner M, Becker BV, Vogel M, Schröder T, Wolfsgruber S, Vasa-Nicotera M, Hammerstingl C, Schwab JO, Thomas D, Werner N, Grube E, Nickenig G, Muller A. Cognitive trajectory after transcatheter aortic valve implantation. *Circ Cardiovasc Interv* 2013;6:615–624.

**8.** Gleason TG, Schindler JT, Adams DH, Reardon MJ, Kleiman NS, Caplan LR, Conte JV, Deeb GM, Hughes GC, Chenoweth S, Popma JJ. The risk and extent of neurologic events are equivalent for high-risk patients treated with transcatheter or surgical aortic valve replacement. *J Thorac Cardiovasc Surg* 2016;152:85–96.

**9.** Haussig S, Mangner N, Dwyer MG, Lehmkuhl L, Lücke C, Woitek F, Holzhey DM, Mohr FW, Gutberlet M, Zivadinov R, Schuler G, Linke A. Effect of a cerebral protection device on brain lesions following transcatheter aortic valve implantation in patients with severe aortic stenosis: The CLEAN-TAVI randomized clinical trial. *Jama* 2016;316:592–601.

**10.** Knipp SC, Kahlert P, Jokisch D, Schlamann M, Wendt D, Weimar C, Jakob H, Thielmann M. Cognitive function after transapical aortic valve implantation: A single-centre study with 3-month follow-up. *Interact Cardiovasc Thorac Surg* 2013;16:116-122.

**11.** Lansky AJ, Schofer J, Tchetche D, Stella P, Pietras CG, Parise H, Abrams K, Forrest JK, Cleman M, Reinöhl J, Cuisset T, Blackman D, Bolotin G, Spitzer S, Kappert U, Gilard M, Modine T, Hildick-Smith D, Haude M, Margolis P, Brickman AM, Voros S, Baumbach A. A prospective randomized evaluation of the TriGuard^TM^ HDH embolic DEFLECTion device during transcatheter aortic valve implantation: Results from the DEFLECT III trial. *Eur Heart J* 2015;36:2070–2078.

**12.** Lansky AJ, Brown D, Pena C, Pietras CG, Parise H, Ng VG, Meller S, Abrams KJ, Cleman M, Margolis P, Petrossian G, Brickman AM, Voros S, Moses J, Forrest JK. Neurologic complications of unprotected transcatheter aortic valve implantation (from the Neuro-TAVI Trial). *Am J Cardiol* 2016;118:1519–1526.

**13.** Orvin K, Dvir D, Weiss A, Assali A, Vaknin-Assa H, Shapira Y, Gazit O, Sagie A, Kornowski R. Comprehensive prospective cognitive and physical function assessment in elderly patients undergoing transcatheter aortic valve implantation. *Cardiology* 2014;127:227–235.

**14.** Schoenenberger AW, Zuber C, Moser A, Zwahlen M, Wenaweser P, Windecker S, Carrel T, Stuck AE, Stortecky S. Evolution of cognitive function after transcatheter aortic valve implantation. *Circ Cardiovasc Interv* 2016;9:e003590.

**15.** Van Mieghem NM, van Gils L, Ahmad H, van Kesteren F, van der Werf HW, Brueren G, Storm M, Lenzen M, Daemen J, van den Heuvel AFM, Tonino P, Baan J, Koudstaal PJ, Schipper MEI, van der Lugt A, de Jaegere PPT. Filter-based cerebral embolic protection with transcatheter aortic valve implantation: The randomised MISTRAL-C trial. *EuroIntervention* 2016;12:499-507.

**16.** Załęska-Kocięcka M, Skrobisz A, Woźniak S, Greszata L, Dąbrowski M, Grabowski M, Piotrowska K, Konopka A, Banaszewski M, Mierzyńska A, Stepinska J. Patterns of changes in functional and neurocognitive status in elderly patients after transcatheter vs. surgical aortic valve replacements. *Minerva Anestesiol* 2018;84:328–336.

**17.** Daniel WW. Biostatistics: A Foundation for Analysis in the Health Sciences. New York: John Wiley & Sons, 1999.

**18.** Naing L, Winn T, Rusli BN. Practical issues in calculating the sample size for prevalence studies. *Arch Orofac Sci* 2006;1:9–14.

**19.** Macfarlane SBJ. Conducting a descriptive survey: 2. Choosing a sampling strategy. *Trop Doct* 1997;27:14–21.
